# Post-Implantation Syndromes After Endovascular Aneurysm Repair: Not Good, But Not Bad Either

**DOI:** 10.1016/j.ejvsvf.2025.04.001

**Published:** 2025-04-09

**Authors:** Andrés Reyes Valdivia

**Affiliations:** Department of Vascular and Endovascular Surgery, Hospital Ramon y Cajal, Madrid, Spain

As the rate of endovascular aortic repair (EVAR) for abdominal aortic aneurysm treatment has clearly increased over the years, descriptions of the treatment complications have been reported by many authors. Typically, endoleaks have been widely discussed and still occupy space in salient discussions regarding best treatment approaches (if necessary). Post-implantation syndrome (PIS) has been described as the systemic inflammation produced by the implantation of the endograft and initially allocated to the “complications box” for EVAR treatment.^1^ These reports on PIS concluded that the importance of PIS was the increased rate of post-operative complications, such as cardiovascular events, longer hospitalisation time, higher re-admission rates and, thus, overall higher costs. In that context, novel devices introduced previously such as the Nellix system (Endologix, Irvine, CA, USA); highlighted the lack of PIS as a major benefit,^2^ although their main clinical benefit was not achieved and finally failed in the endovascular aortic market. Despite these initial findings, and some papers describing some possible aetiopathogenic sources, such as the activation of white blood cells and platelets by endograft material, promoting the release of inflammatory mediators such as cytokines,^3^^,^^4^ there is nowadays clinical consensus regarding the benign nature of PIS after EVAR.^5^^,^^6^

A previous study^7^ that compared 253 patients after standard EVAR (sEVAR) or complex EVAR (cEVAR) concluded that cEVAR procedures were associated with a significantly increased risk of developing PIS compared with sEVAR; however, PIS appeared to have a benign course with no major impact on peri-operative outcomes after cEVAR. The recent paper by Ribeiro *et al.*^8^ describes a single centre experience comparing the findings and implications related to PIS after primary and secondary EVAR. They defined PIS as the combination of fever and elevated C-reactive protein. PIS was a common finding (24.0% in the whole group) and was less frequent in the secondary group (16.5%) than the primary group (32.4%). Importantly, there was no negative clinical impact related to the presence of PIS in the study group. Whether the presence of immunological tolerance plays a role for such findings remains unanswered and might be a matter for future studies with the cooperation of immunologists.

The major limitations of the study were the heterogeneity (as there were so many different secondary procedures) and the rather long hospitalisation time (which allowed for follow up of biomarkers). The authors are encouraged to continue this line of work, as there are not many groups reporting on the importance of PIS. A multicentre study comparing patients with a primary cEVAR and those with a secondary cEVAR may give better understanding into PIS identification and its prognosis significance.

These results, despite the many mentioned limitations, should inform endovascular aortic interventionists about the overall benign course of PIS, which is still less frequent after secondary procedures.

This is of the utmost importance for those centres where fast track EVAR related procedures are established,^9^ carefully explaining to patients that occasionally some degree of fever is to be expected and normal after a primary EVAR procedure, requiring only low doses of non-steroidal anti-inflammatory drugs as symptomatic treatment. Moreover, this will be less expected and less severe for those receiving a secondary procedure. Giving the best quality information for our discharged EVAR patients is essential in order to avoid unnecessary re-admission and explorations, where our current knowledge should drive physicians involved in EVAR into patient individualised follow up. Nevertheless, unusual high and prolonged fever should still raise suspicions about needing to investigate other medical issues that may not necessarily be related to the index EVAR procedure.

## References

[1] Arnaoutoglou E., Kouvelos G., Papa N., Kallinteri A., Milionis H., Koulouras V. (2015). Prospective evaluation of post-implantation inflammatory response after EVAR for AAA: influence on patients' 30 day outcome. Eur J Vasc Endovasc Surg.

[2] Berg P., Stroetges R.A., Miller L.E., Schoefferle J. (2017). A propensity score-matched analysis of inflammatory response with endovascular aneurysm sealing vs endovascular aneurysm repair. J Endovasc Ther.

[3] Martinelli O., Di Girolamo A., Irace L., Baratta F., Gossetti B., Gattuso R. (2020). Post-implantation syndrome: the impact of different devices for endovascular abdominal aortic aneurysm repair and related etiopathogenetic implications. Int Angiol.

[4] Martinelli O., Di Girolamo A., Belli C., Gattuso R., Baratta F., Gossetti B. (2020). Incidence of post-implantation syndrome with different endovascular aortic aneurysm repair modalities and devices and related etiopathogenetic implications. Ann Vasc Surg.

[5] Soares Ferreira R., Oliveira-Pinto J., Ultee K., Voute M.T., Oliveira N.F.G., Hoeks S. (2021). Long term outcomes of post-implantation syndrome after endovascular aneurysm repair. Eur J Vasc Endovasc Surg.

[6] Zlatanovic P., Busch A. (2023). Post-implantation syndrome after complex endovascular abdominal aneurysm repair: not so important after all?. Eur J Vasc Endovasc Surg.

[7] Ribeiro T.F., Soares Ferreira R., Amaral C., Ferreira M.E., Bastos Goncalves F. (2023). Post-implantation syndrome incidence is higher after complex endovascular aortic procedures than after standard infrarenal repair. Eur J Vasc Endovasc Surg.

[8] Ribeiro TF, Soares Ferreira R, Amaral C, Ferreira ME, Bastos Gonçalves F. Post-implantation syndrome incidence after endovascular secondary aortic interventions. EJVES Vasc Forum doi:1016/j.ejvsvf.2025.02.005 [epub ahead of print]. 10.1016/j.ejvsvf.2025.02.005 PMC12270704 40687322

[9] de Donato G., Kontopodis N., Setacci C., Ioannou C.V. (2018). The significance of a fast-track EVAR procedure: it's not the years in your life that count, it's the life in your years. J Endovasc Ther.

